# Metabolomics-Based Investigation on the Metabolic Changes in *Crassostrea gigas* Experimentally Exposed to Galvanic Anodes

**DOI:** 10.3390/metabo13070869

**Published:** 2023-07-20

**Authors:** Nathalie Imbert-Auvray, Denis Fichet, Pierre-Edouard Bodet, Pascaline Ory, René Sabot, Philippe Refait, Marianne Graber

**Affiliations:** 1 UMR 7266 LIENSs, CNRS-La Rochelle Université, 2 Rue Olympe de Gouges, 17000 La Rochelle, France; nathalie.imbert@univ-lr.fr (N.I.-A.); denis.fichet@univ-lr.fr (D.F.); pierreedouard.bodet@univ-lr.fr (P.-E.B.); pascaline.ory01@univ-lr.fr (P.O.); 2 UMR 7356 LaSIE, CNRS-La Rochelle Université, Avenue Michel Crépeau, 17042 La Rochelle, France; rene.sabot@univ-lr.fr

**Keywords:** metabolomics, oysters, corrosion products modelling, sacrificial galvanic anode, zinc, aluminum, biological effects, bioaccumulation

## Abstract

Cathodic protection is widely used to protect metal structures from corrosion in marine environments using sacrificial galvanic anodes. These anodes, either in Zinc, or preferentially nowadays in Al-Zn-In alloys, are expected to corrode instead of the metal structures. This leads to the release of dissolved species, Zn^2+^, Al^3+^, and In^3+^, and solid phases such as Al(OH)_3_. Few studies have been conducted on their effects on marine organisms, and they concluded that further investigations are needed. We therefore evaluated the effects of Zn and Al-Zn-In anodes on oysters stabulated in tanks, under controlled conditions defined through a comparison with those prevailing in a given commercial seaport used as reference. We analyzed the entire metabolome of gills with a non-targeted metabolomic approach HRMS. A modelling study of the chemical species, corresponding to the degradation products of the anodes, likely to be present near the exposed oysters, was also included. We identified 16 and two metabolites modulated by Zn- and Al-Zn-In-anodes, respectively, that were involved in energy metabolism, osmoregulation, oxidative stress, lipid, nucleotide nucleoside and amino acid metabolisms, defense and signaling pathways. The combination of chemical modelling and metabolomic approach, used here for the first time, enlightened the influence of Zn present in the Al-Zn-In anodes.

## 1. Introduction

Cathodic protection (CP) is widely used to protect metal structures from corrosion in marine applications, e.g., ship hulls and propellers, seaport steel sheet pilings, offshore oil platforms, marine renewable energy devices, and so on. CP is commonly applied on marine metal structures using galvanic anodes, a concept first proposed and successfully implemented by Davy in 1824 [1]. Zinc has been a common material for galvanic anodes due to its high efficiency. Nowadays, aluminum-based alloys, and in particular Al-Zn-In alloys, are considered to offer the best performance in seawater [2,3,4].

The protection of seaports structures may require the use of hundreds of tons of galvanic anodes corroding in place of the protected structure metal, mainly carbon steel. This corrosion process then leads to the formation of dissolved species, e.g., Zn^2+^, In^3+^, and Al^3+^, and solid phases, e.g., Al(OH)_3_, which are released in the seaport water. The need to assess the environmental impact of galvanic anodes was then expressed and a few studies were devoted to this problem [5,6,7,8,9]. Very recently, a study was set up over two years (2020 and 2021) to observe whether Al-Zn-In-sacrificial anodes had an impact on the health status of the black scallop *Mimachlamys varia* in a port environment (commercial port and marina of La Rochelle, France), using a multi-biomarker approach [7,10]. The conclusion was that port activities, as well as meteorological conditions, influenced the biomarker results overly significantly and masked the potential effects of these anodes. It was finally suggested that it would therefore be interesting to carry out a similar study in a controlled environment to be free from the influence of port activities and conditions [7]. In 2022, the toxicity of an Al-Zn-In-based galvanic anode on the Pacific oyster, *Magallana gigas* (formerly *Crassostrea gigas*), was studied in controlled conditions. Oysters were exposed for about three months to different concentrations of anode degradation products, obtained with an electrochemical experimental device simulating the dissolution of a galvanic anode. Different biomarkers of the immune system, reproductive parameters, and the metabolic state of the oysters were studied, and the bioaccumulation of metals coming from the anodes was measured. Analyses showed that oysters bioaccumulated Al and Zn and demonstrated some biological effects at the highest concentrations, far above those found in the environment, linked with a possible impairment of immune system and oxidant stress defense at the end of exposure [8]. Further investigations are needed to analyze other potential effects of anodes on marine organisms and to compare Zn- and Al-Zn-In-anodes in conditions closer to exposure levels in port environments. 

Firstly, one of the main concerns for the present study was to estimate what could be the representative conditions prevailing in a seaport. Among parameters defining realistic conditions, the ratio between the mass of galvanic anodes present inside the seaport perimeter and the volume of seawater enclosed in this perimeter seem the most important. As an example, the commercial seaport of La Rochelle (Atlantic coast) was considered. The average volume of water present inside the seaport perimeter was estimated at 5.55 × 10^6^ m^3^ and the overall mass of Al-Zn-In anodes used for the cathodic protection of the steel structures is equal to 135 × 10^3^ kg. The anodes used for the cathodic protection of the La Rochelle seaport steel structures contain a maximum Zn content of 3.0 wt.%, a specific requirement of the seaport managers. The amount of In for this kind of alloy is usually very low, i.e., about 0.02 wt.%, a composition that ensures an active dissolution of the Al matrix [2,3,4]. For these anodes, the specific elements released in the environment are then Al (~97 wt.%), Zn (3 wt.%), and In (~0.02 wt.%). It is expected that the anodes are entirely consumed after 20 years, which, assuming a constant dissolution rate, corresponds to the release of 17.9 kg per day of aluminum, 0.55 kg per day of zinc, and 3.7 g per day of In, for an overall mass of 135 × 10^3^ kg. Considering the overall volume of the commercial seaport of La Rochelle, this mass per day corresponds to Al^3+^, Zn^2+^, and In^3+^ released concentrations of 3.23 µg/L per day, 0.1 µg/L per day, and 0.0007 µg/L per day, respectively.

Following that, to reach further understanding on the environmental impact of galvanic anodes, the present study aimed at determining the effects of Al-Zn-In and Zn anodes on the entire metabolome of oysters with a high level of sensitivity, without any a priori. For that purpose, we chose a non-targeted metabolomic approach using ultra-high performance liquid chromatography coupled to high resolution mass spectrometry (UHPLC-HRMS). In accordance with the results of most recent works [7,8,9], the experimental study was carried out in a controlled environment to evaluate the comparative effects of Zn- and Al-Zn-In-anodes on oysters stabulated in tanks. The Pacific oyster, *M. gigas*, is commonly used for biomonitoring metal contamination because of its great economic interest. Additionally, as it is a filter-feeding mollusk, it has a great capacity to filter large volumes of water and to concentrate environmental contaminants.

The conditions considered for the present study corresponded to a daily released metal concentration that is 25 times higher (Al) or 42 times higher (Zn) to that estimated above for the particular case of the commercial seaport of La Rochelle. A short exposure time (16 days) of oysters to galvanic anodes was then considered, as it was expected to be sufficient to induce detectable effects on oyster metabolism.

Finally, even if Zn-based and Al-Zn-In-based sacrificial anodes may contain various trace elements, e.g., Fe or Cu, the current study was focused on the elements specific to the anodes, i.e., Al, Zn, and In. In the present study, we have included a modelling of the chemical species corresponding to the degradation products of the anodes likely to be present in the vicinity of the exposed oysters. The combination of chemical modelling and metabolomic approach, used here for the first time, gave new information, in particular about the Al-Zn-In anodes mostly used nowadays. 

## 2. Materials and Methods

### 2.1. Experimental Organisms

The experiment was carried out in June 2021, in mesocosm located in a salt marsh in laboratory LIENSs experimental facilities (46°12′13.835″ N; 1°11′43.572″ W).

**Figure 1:**
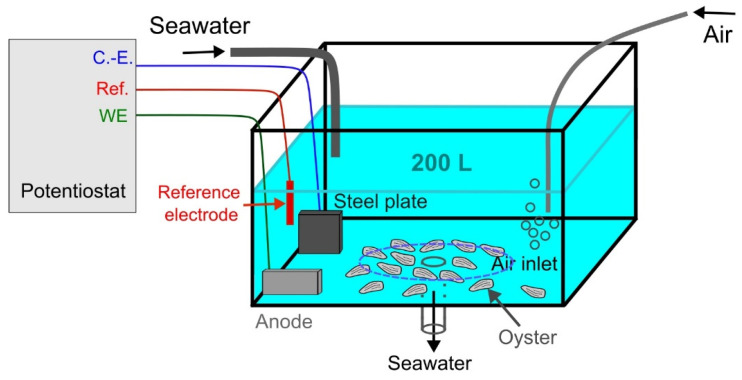
Schematic representation of the experimental setup consisting of potentiostat, galvanic anode, passive sampler, and oysters exposed to the product of anode dissolution in an open-circuit tank. The LSNM-NP passive samplers were set among the oysters, inside the area demarcated by the dashed blue line (C.-E.: Counter-electrode; Ref.: reference electrode; WE: working electrode).

Eighteen-month-old triploid Pacific oysters, *Magallana gigas* (averaged total flesh weight = 22.9 ± 5.9 g, *n* = 100), originated from the same resource, were purchased from a local shellfish farm France Naissain^®^. After eight days of acclimation, oysters were randomly divided into three groups (32 oysters per group) and held in tank (200 L), receiving a constant flow of external water (Figure 1). Each tank was continuously aerated and totally renewed each day with seawater collected from our experimentation salt marsh. Before using, seawater was first decanted in a 16,000 L tank located outside of the lab. Oysters were maintained and fed throughout the experiment (16 days) by the planktonic communities present in the natural sea water of the oyster ponds. The tanks were checked every day to remove the potential dead oysters, but no mortality was detected (temperature 22 ± 2 °C; salinity 37 ± 1‰).

### 2.2. Experimental Setup

Two kinds of anodes were tested as cathodic protection: an anode mainly composed of zinc called «Zn-anode» and an anode mainly composed of aluminum called «Al-Zn-In-anode» (see Section 2.3). 33 oysters were placed on the bottom of each tank (Figure 1): a tank without cathodic protection (control), a tank equipped with the Zn-anode, and a tank equipped with the Al-Zn-In anode. Oysters were exposed for 16 days to the product of anodes dissolution.

Passive samplers (LSNM-NP for cationic metals/DGT^®^ Research) were used to confirm the presence of dissolved metal in water for the anode-equipped tanks and thus show differences with the “control” tank. Four samplers were placed successively in each tank. Each sampler was exposed for four days.

### 2.3. Cathodic Protection

The flow of released matter can be controlled in laboratory experiments via the control of the current flowing through the anode. In this study, a constant anodic current, *I* = 2 mA, was applied using a potentiostat/galvanostat BioLogic SP300 with a three-electrode setup. The working electrode (WE) was the galvanic anode, with a surface of 5 cm^2^ for all experiments. A common Al-Zn-In anode was used, i.e., the Zn content was 5 wt.%. The counter-electrode (C.-E.) consisted of a large carbon steel plate (surface of 125 cm^2^), which thus simulated the protected seaport structure. The reference electrode (Ref.) was an Ag/AgCl/seawater electrode, with a potential *E*_ref_ = +250 mV/SHE. The overall experimental setup is displayed in Figure 1.

The Al-Zn-In anode is mostly made of aluminum, and it can be assumed as a first approximation that the current is mainly associated with the oxidation of Al. Thus, when 1 Al^3+^ ion is produced, three electrons are involved, and the anodic current value of 2 mA ensures the production of 16 mg per day of Al(III) species. Considering the volume of seawater present inside the tank used for the experiments, i.e., 200 L, this production corresponds to a released Al(III) concentration of 80 µg/L per day. This value is 25 times higher than the value estimated as representative of the conditions prevailing in the commercial seaport of La Rochelle. The same ratio applies for the In-released concentration, which was then about 0.017 µg/L per day in our experimental conditions. The Zn amount of the anode used for the experiment was higher than the Zn amount of the anodes used in the seaport of La Rochelle, i.e., 5% vs. 3%, so that the ratio was about 25 × 5/3~42, leading to a Zn-released concentration of 4.2 µg/L per day in our experimental conditions.

As displayed in Figure 1, seawater flowed continuously through the tank so that the overall 200 L volume was entirely renewed after 24 h. The three electrodes were set close to the seawater input, favoring the detachment of the loose white layer of corrosion products (Al(OH)_3_ at temperatures below 70 °C [11]) formed on the anode surface. On the other side of the tank, an air inlet ensured the aeration of the seawater. The studied oysters were placed at the bottom of the tank, around the seawater outlet. It must be noted that the designed laboratory experiment relates to an open system, i.e., the produced Al(III) species may not accumulate inside the tank, although the solid Al(OH)_3_ particles may remain at the bottom of the tank together with the studied oysters. The same situation prevails in a seaport, which is not a closed system either.

Nowadays, it is generally admitted that Al-Zn-In anodes present the best performance in seawater. However, Zn anodes were widely used [2,3], and some remain in service. The experiment was then carried out as described above using a pure (99.9 wt.%) Zn galvanic anode instead of an Al-Zn-In anode. For a Zn anode, when 1 Zn^2+^ ion is produced, 2 electrons are involved. The same current, *I* = 2 mA, was applied to control the dissolution of Zn anodes so that 58 mg per day of Zn(II) species were produced, corresponding to a released Zn(II) concentration of 290 µg/L per day in the tank containing 200 L of seawater.

### 2.4. Chemical Modelling

The corrosion of galvanic anodes in seawater leads to the formation of solid phases. In principle, these compounds do not form an adherent protective layer, because the corrosion of the anode must take place so that CP remains efficient. Depending on the hydrodynamic conditions, solid phase particles are carried away from the anode. The solubility of the corrosion products then governs the maximum local (i.e., close to the solid phase particles) dissolved Al or Zn species concentration (dilution in the overall amount of seawater contained in the seaport of course leads to much lower concentrations). An estimate of this maximum local concentration was determined via a theoretical approach based on a chemical modelling carried out with the PHREEQC Interactive software [12] (version 3.5, 2019) using the PHREEQC Minteq V4 database, derived from MINTEQ A2 version 4 [13,14]. A simplified seawater composition was used for computations and only the main seawater elements were considered. The concentrations, based on the ASTM D1141 standard [15], were as follows (in mmol kg^−1^): [Cl^−^] = 545.9, [Na^+^] = 468.5, [Mg^2+^] = 53.08, [SO_4_^2−^] = 28.23, [Ca^2+^] = 10.28, [K^+^] = 10.21, and [HCO_3_^−^] = 2.3. The temperature was set as 25 °C.

### 2.5. Trace Element Assessments

#### 2.5.1. Sampling and Chemical Analysis of Digestive Gland

32 oysters for each condition were dissected to sample gills (for metabolomic see § 2.1.6) and digestive glands. According to previous studies, digestive gland is the soft tissue in which the highest trace metal contamination occurs for many mollusks including oysters [16,17,18]. This is of particular interest for the detection of less-concentrated metals such as In^3+^. Each digestive gland was carefully dissected out as quickly as possible on ice. Subsequently, the trace elements analysis was performed as described in a previous study [16] using an Agilent 5800VDV ICP-AES and a Thermofisher Scientific XSeries 2 ICP-MS.

A standard certified value sample, DOLT5 (Dogfish liver), was used to validate the analytical method. Average recovery percentages are given relative to the certified values for Al (97% ± 0.07 µg/g Dry Weight) and Zn (101% ± 0.02 µg/g Dry Weight). For indium, no certified organic sample was available.

#### 2.5.2. Chemical Analysis of Passive Samplers

Passive samplers were used to confirm the presence of metals in dissolved form [19] throughout the experiment. Four passive samplers with the reference LSNM-NP open-pore Loaded DGT device for metals (A) in solution (DGT^®^ Research) were successively collected per tank (every four days) to determine total concentrations of Al, Zn, and In (see Section 2.5.2).

The passive samplers were opened in a clean-air environment and the binding phase (Chelex 100 resin gel) was peeled off and eluted with ultrapure nitric acid (1 M). Analyses of Al, Zn, and In were performed with an Agilent 5800VDV ICP-AES and a Thermofisher Scientific XSeries 2 ICP-MS (Thermo Fisher Scientific Inc., Waltham, MA, USA) [7].

#### 2.5.3. Statistical Analysis

Potentially significant differences (*p* < 0.05) in trace elements in both digestive gland and passive samplers were tested between the three conditions (control, Zn-anode, and Al-Zn-In-anode) using the non-parametric Kruskal–Wallis test and PAST software.

### 2.6. Metabolomic Sample Analysis

#### 2.6.1. Tissue Sample Preparation

The gills of two to three oysters per sample were dissected, dried on absorbent paper, and snap-frozen in liquid nitrogen as described in the publication by Ory and collaborators [20]. Thus, a total of 13 control, 13 Zn-exposed, and 14 Al-exposed samples were obtained. The samples were then crushed on ice and adjusted to 1 g. To extract a maximum of compound, each sample was subjected to a triple acetone/acetone/methanol extraction as previously described [21,22]. The three solvent supernatants obtained were then pooled. To remove residual impurities, they were centrifuged at 3000× *g* for 5 min. This total supernatant was recovered and dried under a stream of nitrogen as previously described [21,22]. The dry extract was finally resuspended in 2 mL of 20/80 methanol/water, then diluted ten times in water, centrifuged 5 min at 13,000 rpm, and filtered at 0.2 µm (using low protein binding filter) before MS analyses. The methanol and acetone used were of HPLC grade purity (CARLO ERBA Reagents, Val-de-Reuil, France).

#### 2.6.2. UHPLC/QToF MS Analysis of Samples

An ultra-high performance liquid chromatography (“Acquity UPLC H-class”, Waters, Milford, CT, USA) coupled to high resolution mass spectrometry equipped with an electrospray ionization source was used to analyze the samples (“XEVO-G2-S Q-TOF”, Waters, Manchester, UK). 5 µL of the samples were injected in a column “Acquity UPLC HSST3” (Waters) (2.1 × 150 mm, 1.8 µm), and the products were eluted at a flow rate of 300 µL/min using the same gradient and according to the procedure described in [19]. The analyses were performed in positive and negative ionization modes with MS function in a centroid mode. For the two ionization modes, the MS parameters applied in the ESI source were identical to those used in [19], except that the desolvation gas flowrate was 800 L/h. The instrument was adjusted for the acquisition on a 50–1200 *m*/*z* interval, with a scan time of 0.1 s. To identify ions of interest, targeted MS/MS were achieved using a collision energy ramp varying from 10 to 60 eV depending on molecules. The Leucine Enkephalin (M = 555.62 Da, 1 ng/µL) was used as a lock-mass and the mass spectrometer was calibrated using 0.5 mM sodium formate solution. The samples were analyzed randomly to avoid the effect of possible analytical drift. Analytical repeatability was guaranteed by quality control samples (QC) that were injected every five measurements. The QCs were obtained from the pooling of all samples. Blanks prepared with the last extraction solvent were injected at the beginning and the end of the sample sequence to subtract components from the extraction solvent.

### 2.7. Statistical Analysis

The data were processed as ion peak intensity using the Workflow4Metabolomics (W4M) platform according to the method described by Ory and collaborators [22]. Analytical drift was corrected on the pools using a Loess regression model [23]. Repeatability was assessed through the coefficient of variation (CV) of the QCs. Metabolites with a CV > 0.3 were removed from the analyses. Principal component analysis (PCA) was used to detect natural clustering between samples. Partial least squares-discriminant analysis (PLS-DA) was performed on the log-transformed and Pareto normalized data. The selection of metabolites was based on the importance of their contribution as a predictor variable in the PLS-DA model, as evaluated by their Variable In Projection (VIP). Data with variables in projection (VIP) > 1 can be considered as a metabolite having a significant contribution to the PLS-DA model. The evaluation parameters of the model (R2Y, Q2) were obtained through 7-fold cross-validation, and the permutation test (*n* = 100) was used to measure the effectiveness of the model. This last test consists of keeping the data set constant while randomly permuting the order of the pre-defined variables a set number of times. Student’s *t*-test were then applied to evaluate the significance of differential metabolites, with a rejection threshold of 5%.

### 2.8. Metabolite Identification

After targeted MS/MS of discriminant metabolites, ion spectra corresponding to their fragmentation profiles were uploaded into the identification software Sirius4 [24]. First, the molecular formula determination was achieved by Sirius with C, H, O, N, P, and S as allowed elements, and MS^2^ mass accuracy was fixed at 20 ppm. Next, the tool ‘Predict FPs’ was used to predict the molecular fingerprints of compounds and ‘Search DBs’ was used to search compounds in all proposed structure databases (CSI:FingerID). Finally, ‘CANOPUS’ was used to predict compounds’ class. Molecular formulas were accepted if Sirius scores were >95% and a minimum similarity of 60% was used to restrict the proposed compound structures.

Analytical standards were used and analyzed according to the same method as explained above to check the identification of proline, phenylalanine, and betaine (Sigma-Aldrich, Darmstadt, Germany). Comparison of these standards (retention times, *m*/*z*, and fragments) with the QC validates the identification of these ions.

Finally, we used the classification of Shymanski et al. to support the identification of the metabolites [25]. This method assigns a score to each metabolite based on the degree of confidence in its identification [25].

- Score 1: identification using a standard (same retention times, *m*/*z*, and fragments).
- Score 2a: annotation using fragmentation data from all databases proposed by Sirius with an unambiguous spectrum–structure match.
- Score 2b: the fragments obtained match completely with the proposed structure, which excludes other possibilities, but the data are not completely available in the databases.
- Score 3: proposed annotation of one or more isomeric molecules without the possibility of distinguishing between them because few or no fragments were obtained, or the fragments were common to the different positional isomers.

## 3. Results

### 3.1. Chemical Modelling of Species, Corresponding to the Degradation Products of the Al-Zn-In- and Zn-Anodes

The exact nature and solubility of the corrosion products formed in seawater from Al-Zn-In galvanic anodes is not clearly established yet. As a first approach, a chemical modelling was carried out considering Al(OH)_3_ and Zn(OH)_2_ as the solid phases in equilibrium with the solution. For Al(OH)_3_, two cases were considered that may correspond to two extreme situations. First, amorphous Al(OH)_3_ was chosen as the less stable, i.e., the more soluble solid phase likely to form. Upon ageing, it would tend to a better crystallinity and gibbsite, the most stable Al(OH)_3_ polymorph, was chosen as the less soluble compound likely to form. For zinc hydroxide, only the most stable polymorph, i.e., ε-Zn(OH)_2_, was considered. The results are gathered in Table 1.

Two equilibrium pH values were considered in the pH range of seawater, namely 8.0 and 8.2. The results show opposite trends for the solubility of Zn(OH)_2_ and Al(OH)_3_. As revealed by the decrease of Al dissolved species concentration, the solubility of Al(OH)_3_ decreases when pH decreases from 8.2 to 8.0. Conversely, the Zn-dissolved species concentration increases when the pH decreases from 8.2 to 8.0. Due to the galvanic coupling with the protected steel structure, the oxidation of the anode is faster than the reduction of dissolved O_2_ so that the production rate of metal cations (Al^3+^ and/or Zn^2+^) is higher than the production rate of OH^−^ ions. In other words, the [Al^3+^]/[OH^−^] ratio is higher than 3 and the [Zn^2+^]/[OH^−^] ratio is higher than 2. A decrease of pH is then expected at the vicinity of the anode, as the cations, in particular Al^3+^, are acid species. Consequently, the solubility of Zn(OH)_2_ should be increased by this effect while that of Al(OH)_3_ should be decreased. More detailed chemical modelling studies can be found [26], which show that the lowest solubility for Al(OH)_3_ is reached at pH = 7 and increases significantly for pH values below 5.5 or higher than 8.5 [26].

When dealing with Al(OH)_3_, it is also clearly observed that the ageing of the initial precipitate, that is amorphous Al(OH)_3_, and the subsequent increased crystallinity, would drastically change the solubility of the solid phase. Upon ageing, the dissolved Al concentration in equilibrium with the solid phase would tend to decrease from about 5000–8000 µg of Al per kg of water to 17–26 µg/kg of Al per kg of water for the well-crystallized gibbsite polymorph. In contrast, no such important evolution of crystallinity was reported for Zn hydroxide so that the dissolved species concentration in equilibrium with the solid phase keeps the same order of magnitude, i.e., about 10,000–20,000 µg of Zn per kg of water.

Finally, the main dissolved species are also different when comparing Al and Zn. At both considered pH values, the main Al dissolved species is the Al(OH)_4_^−^ anion, which represents more than 99% of the dissolved Al species. The remaining 1% mainly corresponds to the neutral complex Al(OH)_3_^0^ and to the cation Al(OH)_2_^+^. The modelling also showed that the concentration of the Al^3+^ cation was negligible, i.e., about 10^−12^ mol/kg in any case. For Zn, the main dissolved species is the cation Zn^2+^ (30–35% of the dissolved species). Other important dissolved species are the cation ZnCl^+^, the neutral complex ZnOHCl°, and the anion Zn(SO_4_)_2_^2−^.

To conclude this section, note that these results only give general informative trends as the concentration and nature of dissolved Al and Zn species in equilibrium with the solid phase. They do not depend only on the considered solid phase (as illustrated here for Al) or on pH, but also on temperature, seawater composition, and on the presence and amount of organic matter or other Al-ligands [26] and Zn-ligands.

### 3.2. Levels of Trace Elements

Seawater was continuously renewed from the experimental marsh. The use of passive samplers showed that the water supplied to the tanks contains no detectable quantities of aluminum, indium, or zinc in dissolved form. The analysis of the metal species in their soluble form confirms that in our experimental conditions, aluminum or zinc is the main ion released by Al-Zn-In-anode or Zn-anode, respectively (Table 2). Note that a wide panel of other metals was analyzed, including Fe and Cu present as trace elements in most anodes. The results (not presented) were found to be similar for “control” tank and “anode-equipped” tanks.

Under all conditions, the analysis of aluminum concentration in digestive gland does not show any significant difference (Table 3) after 16 days of exposure. In the same way, digestive glands obtained from oysters exposed to the product released by Al-Zn-In-anode or Zn-anode do not show significant accumulation of zinc compared to control ones, while indium is below the detection values.

### 3.3. LC/MS Data Processing and Analyses

XCMS preprocess from Workflow4Metabolomics platform (W4M) allowed the detection of 1309 and 958 and *m*/*z* features for positive and negative ionization modes, respectively. The data process implied blank removal and batch correction to eliminate instrument signal drift and offset differences between batches. Sample intensities were adjusted using a Loess regression model fitting with the pool values. We evaluated the analytical repeatability of metabolite intensity dataset by calculating coefficients of variation (CV) detected in the Quality Control samples (QC = pool of all samples), and metabolites with CV > 0.3 were removed from the datasets. These different data process steps led to keep 1147 and 871 ions in positive and negative ionization modes, respectively. Multivariate analyses were then performed on these latter datasets.

**Figure 2:**
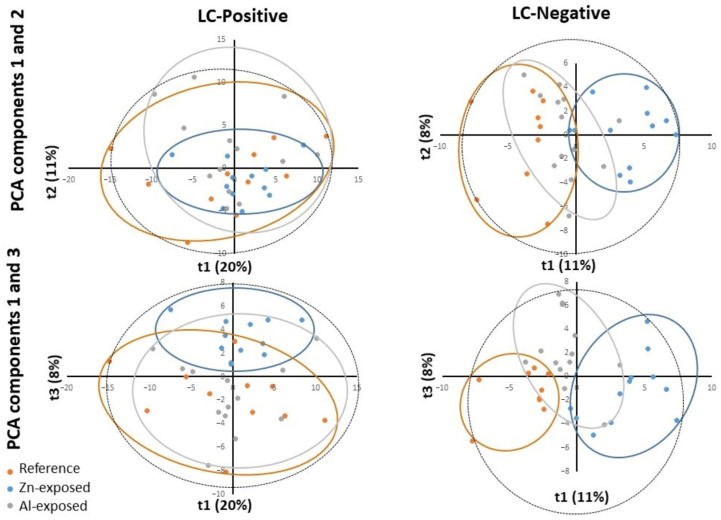
The PCA scores of the oyster samples in each group with the ESI positive (LC-Positive) and negative (LC-Negative) ion modes. T1 represents principal component 1, t2 represents principal component 2, and t3 represents principal component 2. The dotted ellipse represented the confidence limit (95%) of Hotelling’s T2 statistic. The reference samples (“Reference”), the Zn-anode exposed (“Zn-exposed”), and the Al-Zn-In anode exposed (“Al-exposed”) samples are visually grouped in orange, blue, and grey ellipses, respectively.

First, Principal Component Analyses (PCA) showed the sample distribution based on the qualitative and quantitative metabolites composition, highlighting the natural structure of samples (Figure 2).

For LC/MS positive mode, the reference group, Zn-anode, and Al-Zn-In anode-exposed groups did not show obvious trends of separation when considering the two first principal components (PC) of the PCA score plot, PC1, and PC2, explaining 20% and 11% of the total variability, respectively. However, when considering PC3, which explained 8% of the total variability, the PCA score plot described a clustering trend for the reference and the Zn-anode exposed group, but no clustering trend appeared between the Al-Zn-In group and the other groups.

For LC/MS negative mode, variable metabolite presence and intensities, as shown through PC1 and PC2 plot (11% and 8% of the total variability, respectively), induced a clear clustering of reference and Zn-anode exposed groups, while the Al-Zn-In anode-exposed group lay between these two groups with common areas with both groups. When considering PC3 (8% of the total variability), a clustering trend for the Al-Zn-In anode-exposed group from the two other groups appeared.

In summary, on the score plot, the PCA showed a natural clustering between reference and Zn-anode exposed samples for both positive and negative modes, justifying PLS-DA model reliability for both modes. For reference and Al-Zn-In anode groups, the separation appeared only for the negative mode, and PLS-DA was only carried out in this case (Figure 2).

**Figure 3:**
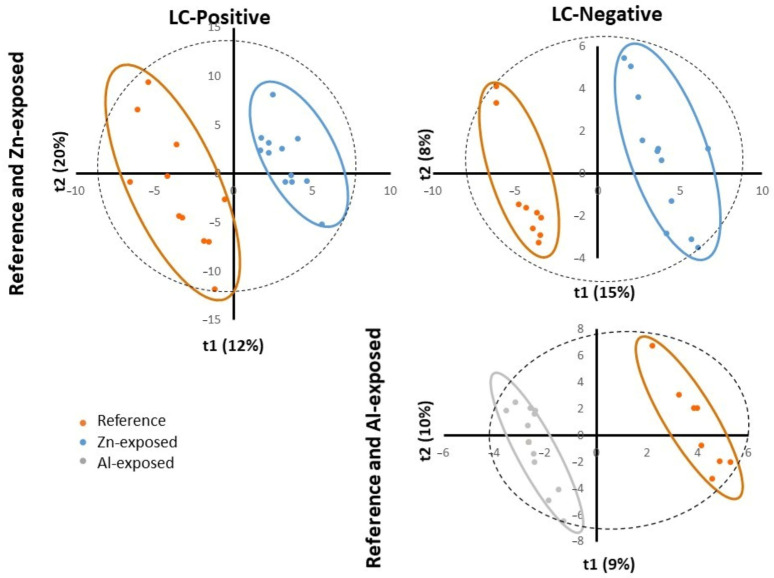
PLS-DA scores plotted for Reference and Zn-exposed samples for ESI positive (LC-Positive) and negative (LC-Negative) ion modes (top) and for Reference and Al-exposed samples for ESI negative (LC-Negative) ion mode (bottom). t1 represents principal component 1, t2 represents principal component 2, and t3 represents principal component 2. The dotted ellipse represented the confidence limit (95%) of Hotelling’s T2 statistic. The reference samples (“Reference”), the Zn-anode exposed (“Zn-exposed”), and the Al-Zn-In anode exposed (“Al-exposed”) samples are visually grouped in orange, blue, and grey ellipses, respectively.

PLS-DA is a supervised method which builds a model that forces the distinction between two firstly defined groups. PLS-DA analyses were first performed to identify metabolites whose abundance was related to exposure to Zn-anode in the two datasets of negative and positive modes. The relevance and performance of the supervised built model was proven with PLS-DA parameters of data consistency (R2) and prediction performance (Q2). R2 (cumulative) reached more than 0.99 for both ionization modes and Q2 (cumulative) reached 0.792 and 0.811 for positive and negative modes, respectively. The permutation test (*n* = 100) and cross-validation test provided *p*-values < 0.05, confirming the consistency of the data and the reliability of the predicted models. Thus, a separation between the reference and Zn-anode exposed groups was significantly demonstrated (Figure 3).

PLS-DA also provides results of variables responsible for forced clustering. Among variables structuring the sample distribution of PLS-DA model, Variable Importance in Projection (VIP) ˃ 1 defined the ones with the significant contribution to the variance between the control and exposed groups. We kept metabolites with VIP ˃ 1 and obtained 255 and 225 metabolites for positive and negative modes, respectively. Among them, 119 and 124 ions for positive and negative modes, respectively, showed a significant difference between the reference samples and the samples exposed to Zn-anode (*t*-test or Mann–Whitney test, *p*-value < 0.05). Among them, 16 metabolites were identified by using one of the identification methods described in the Materials and Methods section.

PLS-DA analyses were then performed to identify metabolites whose abundance was related to exposure to Al-Zn-In anode in the dataset of negative mode. The relevance and performance of the supervised built model was proven with PLS-DA parameters of data consistency (R2) and prediction performance (Q2). R2 (cumulative) reached more than 0.99 and Q2 (cumulative) reached 0.71. The permutation test (*n* = 100) and the cross-validation test provided *p*-values < 0.02, confirming the consistency of the data and the reliability of the predicted models. Thus, a separation between the metabolite composition of the reference and Al-Zn-In anode exposed groups was significantly demonstrated. We kept metabolites with VIP > 1 and obtained 262 ions. To improve the prediction performance of the model and to select the variables presenting the most important contribution in the classification model, a supplementary PLS-DA model was built with only variables with VIP > 1, reaching Q2 = 0.903. This allowed the selection of 82 ions (Figure 3).

Among them, 63 showed a significant difference between the reference samples and the samples exposed to Al-Zn-In anode (*t*-test or Mann–Whitney test, *p*-value < 0.05), but only two were strongly identified.

### 3.4. Metabolites Modulation

#### 3.4.1. Modulations Observed for Zn-Anode Exposed Oysters

**Figure 4:**
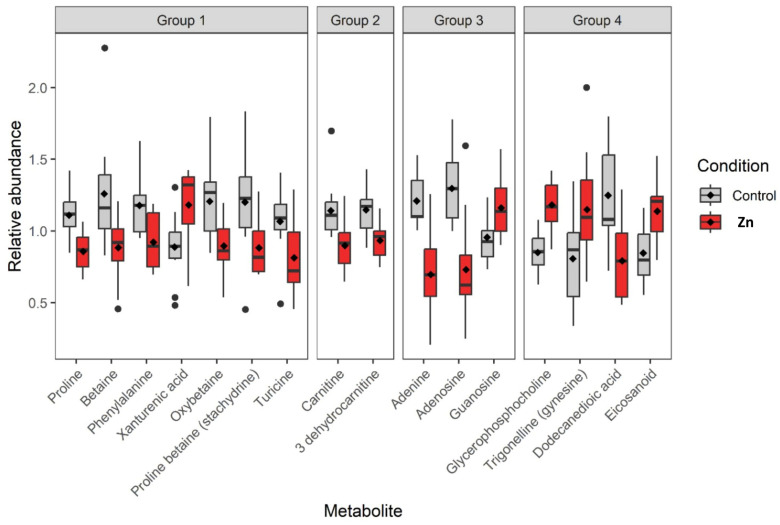
Box plots of the relative abundance of significant compounds (16 identified by the PLS-DA model (Figure 3)) in *M. gigas* gills. Grey plots represent the reference samples, and red plots represent data obtained on oysters exposed to the dissolution of Zn-anode (the mean value is given by the dark diamond-shaped symbol). Metabolites were classified by family: Group 1: Amino acids and derivatives; Group 2: Nucleotides and nucleosides; Group 3: Carnitine acids and derivatives; Group 4: Others.

The use of the different online databases mentioned above or comparison with standards allowed for the confirmed identification of 16 metabolites. Indeed, their scores were less or equal to 2a on the Shimanski scale (Table 4), except for the eicosanoids, a family of compounds for which a more precise annotation was not possible. Among the 16 identified metabolites, three have a confirmed structure (score 1): proline, betaine, and phenylalanine. Among them, four were annotated in negative ionization mode, eight in positive ionization, and four were annotated in both ionization modes (Table 4). They belong to four different biochemical classes: seven are amino acids or derivatives, three are nucleotides or nucleosides, two are carnitine or derivatives, and four are various. These 16 metabolites are significantly modulated between reference and Zn-anode exposed oysters (Figure 4).

The complete list of annotated metabolites is presented in Table 4. Most of them were down-regulated (percentage change of relative abundance; mean ± SD): six amino acids or derivatives (−25.7 ± 2.7%); adenine and adenosine (−34.1 ± 10.5%) for nucleotides and nucleosides group, two metabolites for carnitine and derivatives group (−19 ± 1.9%), and only dodecanedioic acid (−58.6%) for the last group. Some metabolites are also up-regulated, such as xanthurenic acid (in positive mode) and xanthurinate (in negative mode) with a +64.5 ± 44.4% modulation, guanosine (nucleotide and nucleoside group) +21.5% modulation, and two metabolites, and a family of compounds (glycerophosphocholine, trigonelline, and metabolite from the eicosanoids family, 38.7 ± 4.1%) belonging to the last group (“others”).

#### 3.4.2. Modulations Observed for Al-Zn-In Anode-Exposed Oysters

**Figure 5:**
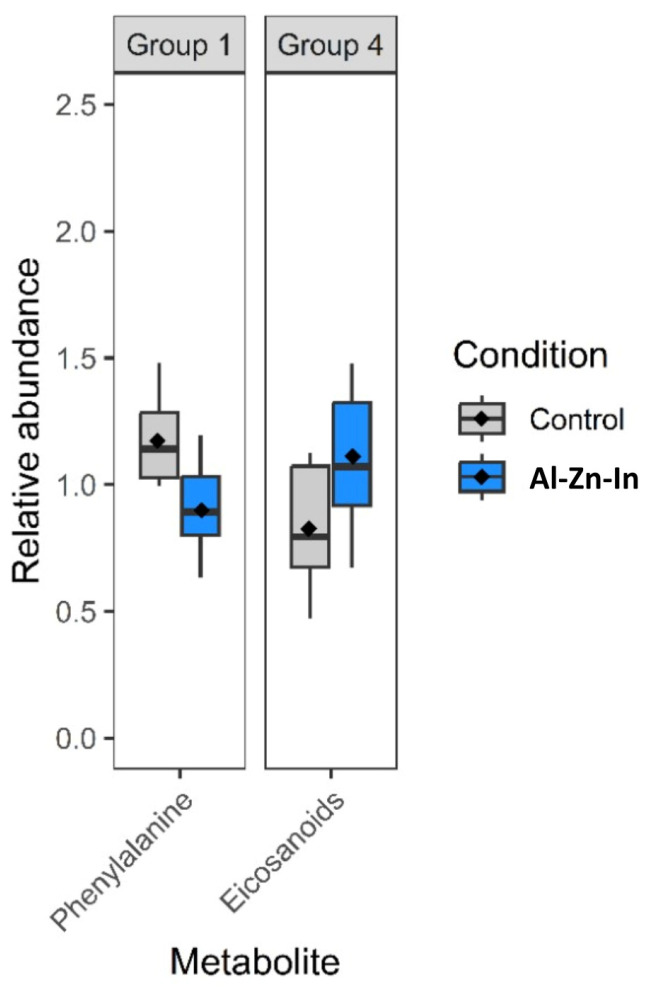
Box plots of the relative abundance of significant compounds (two identified by the PLS-DA model (Figure 3)) in *M. gigas* gills. Grey plots represent the reference samples, and red plots represent data obtained on oysters exposed to the dissolution of Al-Zn-In-anode (the mean value is given by the dark diamond-shaped symbol). Metabolites were classified by family: Group 1: Amino acids and derivatives; Group 4: Others.

The use of the different online databases mentioned above or comparison with standards allowed for the confirmed identification of two metabolites (Table 5) among the 63 ions, showing a significant difference between the reference samples and the samples exposed to Al-Zn-In anode (*t*-test or Mann–Whitney test, *p*-value < 0.05. L-phenylalanine was down-modulated in Al-Zn-In anode-exposed samples compared to reference (−23.3% of relative abundance, *p* = 0.002), whereas the metabolite from eicosanoid family is up-regulated (+34.6%, of relative abundance *p* = 0.002) (Figure 5). Among the 61 remaining ions, 31 were also present in the list of ions showing a significant difference between the reference samples, and the samples were exposed to Zn-anode but could not be identified. Thirteen were also present in the list of ions, showing a difference between the reference samples and the samples exposed to Zn-anode, but not significantly, and only 18 ions were specific to Al-Zn-In anode.

## 4. Discussion

To our knowledge, the present study is the first study to use a metabolomic approach to evaluate the effects of metals released by galvanic anode on oysters and combining this metabolomic approach with a chemical modelling study of degradation products from these anodes. The comparative effects of Zn- and Al-Zn-In- anodes were carried out under experimental conditions without sediment and with an important water renewal. Gills were chosen as target organs, as in bivalves they form the main interface between the organism and the surrounding water and have therefore become a key organ for food absorption. Given the high-water filtration rates of bivalves, gills also constitute a significant pathway of incorporation of pollutants and, among them, metals via seawater [27]. It has been shown that gills would have a high defense ability against contaminants, with a notable presence of antioxidant enzymes and metallothioneins in particular [28]. Consequently, gills play a central role in scenarios of acute exposure to metals by integrating both absorption and metabolism.

### 4.1. Chemical Modelling and Its Implications

The computed dissolved species concentrations given in Table 1 correspond to the equilibrium conditions between the considered solid phase and the solution. Such concentrations would be reached in the immediate vicinity of the anode, inside the porous layer of corrosion products surrounding the metal, and (maybe) a few centimeters away from the anode surface. They correspond to a maximum value and the dissolved species concentration is expected to decrease with the distance from the anode down to the low value that is typical of bulk seawater. The concentration profile should mainly be linked to the hydrodynamic conditions.

Al and Zn could also be carried away from the anode as fragments of the solid phase. Such solid particles may accumulate locally in the environment, more likely in confined areas where stagnant conditions are met, and solid particles falling from the anode could accumulate in the seabed below. The subsequent dissolution of these particles would then lead to a local enrichment in Al and/or Zn-dissolved species. In the experimental conditions considered here (Figure 1), the oysters were scattered around the water outlet of the tank. The solid particles detaching from the corrosion product layer covering the anode were then carried away by the seawater flow towards the oysters.

In the case of Al-Zn-In anodes, both Al and Zn ions are released in the solution. The Al- and Zn-dissolved species concentrations are controlled by the solubility of the respective solid phases (Table 1). Consequently, due to the higher solubility of Zn(OH)_2_ with respect to Al(OH)_3_, the dissolved Zn species concentration at the vicinity of an anode could be higher than the Al dissolved species concentration, even though the anode contains only 3–5 wt.% Zn and is mainly composed of Al. The main Al-dissolved species coming from galvanic anode dissolution is the Al(OH)_4_^−^ anion, which represents more than 99% of the dissolved Al species at the pH of seawater. For Zn, the main dissolved species is the cation Zn^2+^ (30–35% of the dissolved species), with other important dissolved species being the complex ions ZnCl^+^, ZnOHCl°, and Zn(SO_4_)_2_^2−^. It is not possible to accurately assess the amounts of soluble Al and Zn species to which oysters are exposed, as solid Al and Zn particles may accumulate near the oysters and subsequently dissolve. However, as mentioned earlier, there could be a higher level of exposure to Zn^2+^ ions than to Al(OH)_4_^−^ ions when using Al-Zn-In anodes.

In the case of Zn-anode, it can be stated that the quantities of Zn^2+^ ions to which oysters are actually exposed to are higher than in the case of Al-Zn-In anodes, higher than the sum of the individual ions coming from the latter anode. This would result from the higher amount of Zn(OH)_2_ particles produced and released in the environment, and from the relatively high solubility of Zn(OH)_2_.

However, the quantities of Zn^2+^ and Al^3+^ ions to which bivalves are exposed when using galvanic anodes are much lower than the corresponding quantities afforded from sulphate salts. This is consistent with a study performed in 2010, showing that the dissolution of Al and Zn from a galvanic anode was less toxic for the urchin *Paracentrotus lividus* than sulphate salts [5].

### 4.2. Bioaccumulation

The use of passive samplers confirms the higher quantity of dissolved form of aluminum or zinc in tanks equipped, respectively, with Al-Zn-In anodes and Zn anode compared to the control tank. For indium, it could not be detected. However, neither oysters exposed to the corrosion of Zn-anode nor those exposed to the corrosion to Al-Zn-In anode accumulate Zn or Al in their digestive gland. Caplat et al. (2012) showed that the digestive gland of *M. gigas* has a great capacity to accumulate zinc released by a sacrificial anode made of Zinc [29]. However, according to their experimental design, the concentration of zinc to which their oysters were exposed was much higher than in our conditions—between seven and more than 100 times higher. Under their conditions, the digestive glands of oysters exposed to the product of sacrificial anode exhibited an increase in zinc concentration after 21 days of exposure, when the external nominal zinc concentration was about 0.3 ± 0.04 mg/L and after 96 h when the external nominal zinc concentration was about 10.2 ± 1.2 mg/L [29].

Concerning the bioaccumulation of degradation products from anodes mainly composed of Al, very few studies were performed on marine bivalves.

In the first study, the concentration of aluminum was measured in the digestive gland of mussels, *Mytillus edulis*, exposed to sacrificial anode composed mainly of aluminum (93.2% min aluminum, 2.73 kg/dm density) [30]. Seawater was continuously renewed, and the nominal aluminum concentration fluctuated between 270 and 810 µg/L in their experimental conditions. At the maximum, obtained on the 13th day of contamination, they found that digestive glands of exposed mussels concentrated about four times more Al than non-exposed ones and six times more Al than the total soft tissues. Our experimental conditions exposed our oysters to a much lower level of aluminum (80 µg/L of Al^3+^ released from the anode). After 16 days of experimentation, the digestive glands of oysters exposed to Al-Zn-In anode did not show any bioaccumulation of Al^3+^, nor Zn^2+^ or other metal cations such as In^3+^.

In a second study, Levallois et al. worked on the bioaccumulation of Zn and Al in oysters after an 84-day exposure to Al-based anodes, such as our Al-Zn-In anodes [8]. They found that bivalves bioaccumulated more zinc than aluminum (in total tissues), even if nominal aluminum concentrations during exposures were higher. Moreover, exposure time did not influence the bioaccumulation of aluminum in contrast to zinc. Al presented close and lowest bioconcentration factors (BCF) values, whereas Zn BCF values increased in relation to the decrease of exposure concentrations [8]. BCF measures the ability of an organism to bioconcentrate an element in its tissue considering the concentration of that element in the water. If we want to compare our Al and Zn bioaccumulation results in the presence of Al-Zn-In anodes with those of these authors, under conditions most like ours, we must consider their results after 7 and 29 days for nominal Al and Zn exposures of 65 μg/L and 15 μg/L, respectively, on the one side, and 125 μg/L and 22 μg/L on the other side. Their results showed an approximate doubling of Al levels and a slight increase in Zn concentrations in total flesh compared to controls, whereas we did not observe any differences in the digestive glands after 16 days of exposure to 80 µg/L Al and 4.2 µg/L Zn.

In the present study, the target organ for bioaccumulation is the digestive gland. The digestive gland is a detoxification organ, meaning that metals are taken up, sequestered, and transformed into less toxic forms. In our study, no accumulation of any targeted metal was observed after 16 days of exposure. The main reasons could be the relatively weak nominal concentrations of product degradation anodes in our conditions and the insoluble nature of aluminum, combined with the fact that the gills capture mainly minerals in soluble form, when performing their feeding function. This is not the case for grazer species such as limpets (*Patella vulgata*), which are grazers that are able to ingest insoluble species. Therefore, it could be interesting to carry out future studies to compare species with different feeding behaviors.

### 4.3. Ecotoxicological Effects of Zn- and Al-Zn-In Anodes on Marine Organisms Assessed by Other Methods Than Metabolomics

For now, many studies focused on ecotoxicological effects of Zn- and Al-Zn-In anodes on marine organisms using classical methods with biomarkers and assays at the cellular level, rather than untargeted metabolomic methods.

In 2018, Kirchgeorg et al. [31] published a review about emissions from offshore corrosion protection systems and their potential effects on the marine environment. The authors mentioned that a few studies investigated the fate and the environmental effects of products emitted by the galvanic anodes. They concluded that the potential ecotoxicological effects of the different species formed from Al or Zn (hydroxides or complex) still needed further investigation to be elucidated. The data of indium effects in the marine environment are rare [30]. The toxicological effects of In on freshwater swamp shrimp (*Macrobrachium nipponense*) was studied and a median lethal concentration for indium (III) between 6.9 and 21.5 mg/L (LC50) was found [32].

Concerning the effect of Zn-anodes, in 2012, Muttin et al. monitored the effects on the Pacific oyster *Magallana gigas* of the degradation products of a sacrificial anode with a minimum of 99.31% zinc, reproducing the same chemical forms of zinc (II) released from anodes as in natural conditions [6]. They performed chronic exposure with 0.5 mg/L Zn for 10 weeks and acute exposure with 10 mg/L Zn for 7 days and analyzed both gills and digestive glands. Both types of exposures led to a decrease of circulating hemocytes and an increase of metallothioneins and mRNA expression, among others [6]. These results showed that several biological functions were affected by exposure to Zn-anodes, as appeared in our metabolomic results (see below). However, they correspond to much higher levels of zinc exposure than ours: twice the nominal concentration for long-term exposure and 34 times higher for short-term exposure.

Concerning the effect of anodes containing Al as a major component, their toxicity was studied on the Pacific oyster, *M. Gigas*, in controlled conditions in 2022 [8]. Oysters were exposed for about three months to different Al concentrations obtained with an electrochemical experimental device simulating the dissolution of a galvanic anode. Total Al nominal concentrations were equal at the maximum to 300 μg/L, i.e., at concentrations approximately four times higher than in the present study and for a longer period [8]. After 84 days, phagocytic efficiency decreased by 40% and changes in the number or size of lysosomes were observed at this Al concentration, which may indicate a weakened immune system. Moreover, the oysters presented significantly lower levels of malondiadehyde, an indicator of lipid peroxidation, which may be explained by antioxidant defense [8]. Again, several biological functions appeared affected upon exposure of products from sacrificial anodes, in this case Al-based anodes.

These different results demonstrate that the Pacific oyster *Magallana gigas* is sensitive to products emitted by both Zn and Al-Zn-In anodes, with some biological effects observed at the highest metal concentrations tested. These initial results prompted us to continue studying the mechanism of action of anode products on oyster metabolism, at doses closer to those encountered in the environment and on a molecular scale. In the following paragraphs, we discussed the effects of the two types of sacrificial anodes on the different biological functions, as observed with the help of metabolomics, following an untargeted and most sensitive analysis for nominal concentrations closer to those observed in port conditions. We have not attempted to make a link with these initial results. Indeed, we think that it would be illusory to try to link our results obtained using a molecular approach with those obtained previously under other conditions using a cellular approach.

### 4.4. Ecotoxicological Effects of Zn- and Al-Zn-In Anodes on Marine Organisms Assessed by Metabolomics in the Present Study

As mentioned above, the quantities of Zn^2+^ ions to which oysters are actually exposed are higher in the case of Zn anodes than the total quantity of ions coming from Al-Zn-In anodes. Moreover, there could be a higher level of exposure to Zn^2+^ ions than to Al(OH)_4_^−^ ions when using Al-Zn-In anodes. This explains why we found many more metabolites impacted by Zn-anode exposure than by Al-Zn-In: 119 and 124 ions from mass spectrometry analysis for positive and negative modes, respectively, showed a significant difference between the reference samples and the samples exposed to Zn-anode, whereas only 63 showed a significant difference between the reference samples and the samples exposed to the Al-Zn-In anode. The latest comparison was analyzed in negative mode only, as the two groups could not be clearly separated in positive mode. This also explains why the two metabolites identified as being modulated by Al-Zn-In anode exposure are also among the 16 metabolites modulated by Zn-anode. Thus, it could be that the effects observed in the presence of the Al-Zn-In alloy anodes are probably mainly due to Zn^2+^ ions. An additional argument in favor of this hypothesis is that, among the 61 remaining ions, showing a significant difference between reference and A-Zn-In anode exposed oysters, 31 were also present in the list of ions showing a significant difference between the reference and the Zn-anode exposed samples, but they could not be identified. Further, as illustrated by the low number of identified ions, the identification of metabolites remains a challenging task, particularly with poorly characterized species such as *Magallana gigas.*

Below, we discuss the potential effects of the identified metabolite modulations on several biological functions in which they are involved.

#### 4.4.1. Energy Metabolism

Among the metabolites impacted by both Zn- and Al-Zn-In anodes, we found the down-regulation of L-phenylalanine. One hypothesis that cannot be ruled out is that this phenylalanine drop is due to a disturbance of the feeding caused by the induced stress. Nevertheless, it can also be speculated that it may be an indirect effect of energy and lipids metabolism alterations induced by anode degradation product exposure. Indeed, the exposure to Zn^2+^ or Al^3+^ may lead to an energy overdemand due to the need to develop protective mechanisms for the cells, such as overproduction of metallothioneins, glutathione, molecular chaperones, and antioxidant pathways [33,34,35]. Furthermore, osmoregulation phenomena, which are overstressed in the presence of metal contaminants, are energetically costly processes, and the maintenance of ion gradients is one of the most ATP-consuming processes [36]. L-phenylalanine is an amino acid that is both glucogenic and ketogenic, and it can be consumed under energy-deficient conditions in aquatic invertebrates in the place of carbohydrates or lipids used in normal circumstances [36]. Moreover, mitochondrial efficiency and coupling were shown to be reduced and proton leak was elevated after exposure to toxic metals such as cadmium, copper, zinc, and mercury in marine organisms [36]. In the same way, Meng et al. observed that Zn affected the tricarboxylic acid cycle by influencing the expressions of Fe-containing proteins and disrupted the electron transport chain by inhibiting complex I–IV related protein expressions. This was noted in oysters after nine days of exposure with nominal Zn concentration of 30 μg/L and analysis of gills by proteomics [37]. More recently, we found a decrease in L-phenylalanine in scallops exposed to Zn^2+^ (150 μg/L), after metabolomic analysis of their gills [20]. As L-phenylalanine is an essential amino acid for bivalves, this decrease may have different biological effects linked among others with impaired protein synthesis.

Concerning the up-modulation of glycerophosphorylcholine observed in our study upon Zn-anode exposition, this could also be linked to energy metabolism disturbances. This compound is a phosphocholine precursor, as it can be converted into choline and glycerol 3-phosphate by glycerophosphocholine phosphodiesterase. ATP and choline are converted into phosphocholine and ADP by choline kinase [38]. Recently, Ramirez et al. also observed an up-modulation of glycerophosphocholine in Mediterranean mussels (*Mytilus galloprovincialis*) exposed to a 10 µg/L nominal concentration of the antidepressant venlafaxine and interpreted it as an overdemand in phosphocholine following an inhibition in the conversion of choline and ATP into phosphocholine and ADP [39].

#### 4.4.2. Osmoregulation

The down-modulation of L-phenylalanine observed in both Zn- and Al-Zn-In anodes exposed oysters may also be related to osmoregulation phenomena. Indeed, L-phenylalanine is a precursor of catecholamines, and its lowered level can lead to a decrease in dopamine. The latter molecule is, along with serotonin, a neurotransmitter involved in the modulation of Na^+^ and K^+^ transport in crustacean gills [40] and in the lateral cilia of bivalve gills [41]. The down-modulation of L-phenylalanine and, consequently, dopamine could therefore have an impact on osmoregulation in gill cells of oysters exposed to both types of anodes. In the present case, we did not observe a concomitant decrease in dopamine, which does not allow us to confirm this hypothesis. However, it cannot be ruled out, as it is not uncommon in metabolomics to see variations in a single metabolite in a pathway affected by a stressor.

A link between the decrease of L-phenylalanine and osmoregulation has been suggested previously [20,42], after 48 h of exposure to zinc, respectively, ZnCl_2_ (20, 50, 100 and 150 μg/L) for the clam *Ruditapes decussatus*, and Zn^2+^ (150 μg/L) for the variegated scallop (*Mimachlamys varia*). In the first case, the phenylalanine decrease was associated with a significant increase in the production of organic osmolytes (hypotaurine and homarine) and a decrease in the free amino acid content.

Modulations of proline, proline betaine, betaine, and trigonelline in Zn-anode-exposed oysters are also related to osmoregulation [43,44,45]. These molecules affect the ionic strength of the cytosol and thereby maintain osmotic pressure within the cell. Proline was identified as one of the main osmolytes in oyster *Crassostrea virginica* and more generally free amino acids, among them proline, predominantly contribute to the intracellular pool of osmolytes in all the investigated molluscan species [44]. Proline betaine was identified in *Staphylococcus aureus* bacteria as a highly effective osmoprotectant [43] and as an increasing metabolite in diatoms in response to long-term salinity [46]. Betaine and trigonelline are known as organic osmolytes in marine bivalves. Betaine was found to increase in oysters *Crassostrea hongkongensis* after 6 months of exposure to metal pollution with a dose-response effect [47] and, on the contrary, decreased in male oysters *Crassostrea hongkongensis* following *Vibrio harveyi* infection [48]. In female oysters, trigonelline was found to decrease following *Vibrio harveyi* infection [48]. Trigonelline also varies in *Mytilus galloprovincialis* before and after immune stimulation with *Vibrio splendidus* [49]. In the present study, we observed a down-regulation of betaine in oyster gills after 16 days of exposure to Zn-anode. In a study performed in metal polluted estuaries in Southern China, in which Cu and Zn were the major contaminants, Jiu et al. also observed a decrease of betaine in oyster *Crassostrea hongkongensis* gills in animals exposed to pollution compared to oyster gills from a clean site. They interpreted it as a disturbance of osmotic regulation in gills induced by metals [50]. Thererfore, it seems that these organic osmolytes can vary up or down, depending on the different conditions under which the different stressors are applied in combination with other different environmental factors.

The increase in glycerophosphorylcholine upon exposure to Zn-anode could also be attributed to an osmoregulatory mechanism if we refer to the role played by this molecule in humans. Indeed, glycerophosphorylcholine is one of the four majors organic osmolytes in renal medullary cells, changing their intracellular osmolyte concentration in parallel with extracellular tonicity during cellular osmoadaptation. Kidneys (especially medullar cells) respond to hypertonic stress by accumulating the organic osmolytes glycerophosphorylcholine, betaine, myo-inositol, sorbitol, and free amino acids. Glycerophosphorylcholine is formed in the breakdown of phosphatidylcholine [51].

L-carnitine, which is down-modulated in our study in the presence of Zn-anode, has also been described as an organic osmolyte in bacteria and archaea [52].

Finally, a molecule identified as an eicosanoid was up-regulated in both Zn- and Al-Zn-In anode-exposed oyster gills. This molecule may also have a role in osmoregulation. Eicosanoids correspond to a group of molecules, including prostaglandins and related oxygenated metabolites of certain C20 polyunsaturated fatty acids. In addition to many other roles, they are involved in salt and water transport in epithelial tissues in bivalves, leading to the release of osmotically active species and the restoration of normal cell volume [53]. In a study on the influence of salinity on the metabolism of the Pacific oyster *M. gigas*, it was shown that acclimation to salinity involved a major remodeling of membrane fatty acids. The level of arachidonic acid (20:4n-6) varied linearly with salinity, likely reflecting its mobilization for prostaglandin synthesis [53].

#### 4.4.3. Oxidative Stress

Metal pollutants can disrupt mitochondrial function in intertidal bivalves [54], and mitochondria are one of the main sources of ROS due to electron leakage in respiration [55]. This hypothesis is reinforced by the fact that a decrease in carnitine is simultaneously observed, and that this metabolite was proposed to be a marker of mitochondrial activity after a (^1^H NMR)-based metabolomic analysis to *Crassostrea virginica*, investigating the differences in the metabolic profile of different organ groups [44].

The decrease in carnitine observed here may also be related to its consumption to play its antioxidant role. The antioxidant defense system is mainly composed of three enzymes: glutathione peroxidase, catalase, and superoxide dismutase. L-carnitine can protect these enzymes from further peroxidative damage [56]. The role of carnitine as an antioxidant against lipid peroxidation and against deleterious effects of ROS has already been mentioned in a study discussing of the adverse effects of wastewater effluent on the damselfly larvae (*Coenagrion hastulatum*), a common aquatic invertebrate species [57].

#### 4.4.4. Lipid Metabolism

L-carnitine was found to be down-modulated in the gills of oysters exposed to Zn-anode. The main function of this compound is the transfer of long-chain fatty acids, in the form of acylcarnitine, and to mitochondria for subsequent β-oxidation for energy production. In addition to its role in energy production, carnitine conjugation decreases the number of acyl residues attached to coenzyme A (CoA) and plays a key role in maintaining the homeostasis of the mitochondrial acyl-CoA/CoA ratio [58]. Therefore, the L-carnitine decrease in presence of zinc may be related with both abnormal fatty acid metabolism and mitochondrial dysfunction. In this sense, as we already mentioned above, Tikunov proposed that carnitine may be a useful marker of mitochondrial activity after the metabolomic analysis of *Crassostrea virginica* in different organ groups [44].

The process of long-chain fatty acids transport, known as carnitine shuttle pathway, was found to be disturbed in several studies concerning invertebrates exposed to pollutants, often with changes in acylcarnitine profiles rather than a decrease in carnitine, as is the case in the present study. For example, diclofenac was found to affect the carnitine shuttle pathways in *Hyalella azteca*, a freshwater invertebrate, at environmentally relevant concentrations, showing the decrease of three different acylcarnitine [59]. In another recent study, three different acylcarnitine were up-regulated in damselfly larvae (*Coenagrion hastulatum*) after wastewater effluent exposure [57]. We observed changes in acylcarnitine profiles when scallops were exposed to different stressors in laboratory conditions: an increase of seven different acylcarnitine metabolites at the end of the two-hour emersion periods [22], a down-regulation of three after 48 h exposure to a nominal zinc concentration of 150 μg/L [20], and up-regulation of one under 48h exposure to a copper concentration of 82 µg/L) [60]. All these observations concern situations found in the environment, and it therefore seems that the carnitine shuttle pathway is frequently impacted by various stressors: pharmaceutical residues, metallic pollutants, or anoxia.

It should be added that carnitine also functions as a scavenger by binding acyl residues that from the intermediary metabolism of amino acids, helping in their elimination. This mechanism is essential in binding/removing abnormal organic acids and is likely to cause carnitine deficiency [58].

3-Dehydrocarnitine, an intermediate in carnitine degradation, was also found to decrease in the present study, and in gills of Zn-anode-exposed oysters. This metabolite was found to be significantly dysregulated in a study about the possible adverse effects of fadrozole, a molecule used in breast cancer medication, on the adult freshwater mussel *Lampsilis fasciola* [61].

Dodecanedioic acid was found to be down-modulated in Zn-anode-exposed oysters. This metabolite is a dicarboxylic acid, which can be rapidly oxidized in the peroxisomes and then transferred to the mitochondria for further degradation. It might be derived from long monocarboxylic acids through an initial ω-oxidation followed by β-oxidation [62]. Thus, the observed decrease of dodecanedioic acid could indicate peroxisomal dysfunction.

#### 4.4.5. Nucleotide and Nucleoside Metabolism

Both adenine and adenosine decreased here in the presence of Zn-anode. Adenine, a purine derivative, forms adenosine, a nucleoside, when attached to ribose and deoxyadenosine when attached to deoxyribose. Adenine is therefore one of four nitrogenous bases at the origin of nucleic acid (both RNA and DNA) synthesis. Adenosine can be bonded with one to three phosphoric acid units, yielding the energy carriers adenosine mono-, di-, and triphosphate (AMP, ADP, and ATP). Adenosine also plays a role in signal transduction as a cyclic adenosine monophosphate (cAMP). Guanosine was found to be up-modulated in the presence of Zn-anode. This purine derivative is also involved in various biochemical processes, including the synthesis of nucleic acids such as RNA and intracellular signal transduction. This last role is played by cyclic guanosine monophosphate (cGMP), which is one of the phosphorylated forms of guanosine, with guanosine monophosphate (GMP), guanosine diphosphate (GDP), and guanosine triphosphate (GTP).

Adenine, adenosine, and guanosine are therefore essential for life and their metabolic relationships with many metabolic pathways are strong, which makes it difficult to interpret their modulation. It may correspond to the restriction of substrates involved in DNA and RNA turnover and repair, the overdemand of energy carrier to develop detoxification processes, and the inhibition of key enzymes involved in purine and synthesis or altered phosphagen metabolism. The impact of pollution on nucleotide metabolism of marine mollusk or fish has often been mentioned [60,63,64,65].

We observed a disordered nucleotide metabolism in scallops exposed to Zn, manifesting by a lower level of xanthine [20]. Indeed, in the catabolism of purine nucleotides, adenosine monophosphate (AMP) and GMP both lead to xanthine.

In a study of the effect of pharmaceutical active compounds on *Danio rerio* fish antibiotic exposed for 72 h, it was found that the main affected metabolic pathway was related to purine metabolism, especially guanosine [66]. The authors mention that this compound has been shown to be involved in protecting neurons against excitotoxic damage in vertebrates, but its role in invertebrates has not been fully explored yet.

#### 4.4.6. Amino Acids Metabolism

Xanthurenic acid, which is a metabolite from tryptophan catabolism, was up-regulated in the gills of Zn-anode exposed oysters, indicating that the tryptophan metabolism was disturbed. Tryptophan is not only essential for protein biosynthesis but also serves as a precursor to serotonin, a main actor in the neuroendocrine–immune regulation in marine bivalves [67].

#### 4.4.7. Defense or Signaling Pathways

Turicine (cis-4-Hydroxy-D-proline betaine), also known as combretin A, was found to decrease in the gills of oysters exposed to Zn-anode. This proline derivative is a secondary metabolite. As such, it may serve as defense or signaling molecule, or it may simply be a molecule that arises from the degradation of other secondary metabolites.

Eicosanoids that have already been mentioned for their role in osmoregulation have also been shown to be important cellular signaling molecules related to inflammation and immune regulation in invertebrates [68]. A molecule identified as an eicosanoid was up- regulated in both Zn- and Al-Zn-In anode-exposed oysters.

## 5. Conclusions

Our study demonstrates the early effects of exposure of oysters to the degradation products of both types of sacrificial anodes used to protect marine metal structures from corrosion: Zn and Al-Zn-In anodes. These effects were observed with released Al and In concentrations that were 25 times higher than those expected in the seaport used as a reference (La Rochelle commercial seaport, Atlantic coast, France), and 42 times higher in the case of Zn.

These effects are mainly observable for oysters exposed to the Zn-anode when the digestive gland has not yet accumulated zinc. For this first type of anode, the main dissolved degradation product is Zn^2+^. For Al-Zn-In anodes, our chemical modelling study indicates that the main dissolved species among degradation products is Al(OH)_4_^−^. But even though Al is the overwhelmingly majority constituent and Zn is a very minority constituent of anodes, there could be a higher level of exposure to Zn^2+^ ions than to Al(OH)_4_^−^ ions due to the solubility properties of both elements. Thus, the effects observed in the presence of the Al-Zn-In alloy anodes could mainly be due to Zn^2+^ ions, hence the interest in limiting the percentage of zinc in this type of alloy to a minimum. However, the effects on oyster metabolism seem to be less pronounced than for the Zn-anode, which is an important finding of this study.

No detectable effect attributable to indium was observed in our experimental conditions. It must be recalled that this element is present as traces (~0.02 wt.%) in Al-Zn-In anodes.

As mentioned above, only a few ions were identified, mainly due to the low characterization of the studied species. We queried all databases offered in the Sirius software. Unfortunately, many ions remain unidentified, affecting our reading of metal exposure consequences on oysters. Therefore, structure databases need to be completed to make metabolite identification easier.

One of the strong points of this study was to combine chemical modelling of the degradation products of both sacrificial Zn- and Al-Zn-In anodes, with a metabolomic approach, to identify the effects of these anodes on the metabolism of the oyster in an untargeted way. We studied the exposure of a cocktail of products, soluble or not, on the oyster, and not the effect of a metal in a single cationic form.

## Figures and Tables

**Table 1:** Chemical modelling: dissolved species concentrations computed for two pH values, corresponding to the equilibrium conditions with the considered solid phase (concentrations given in µg/kg), main dissolved species, and relative abundance (R.A.) and a list (not exhaustive) of other dissolved species with corresponding R.A.

Equilibrium Solid Phase	Amorphous Al(OH)_3_	Amorphous Al(OH)_3_	Gibbsite	Gibbsite	ε-Zn(OH)_2_	ε-Zn(OH)_2_
pH	8.0	8.2	8.0	8.2	8.0	8.2
Dissolved species conc.	5153	8410	17	26	21582	9614
Main dissolved species	Al(OH)_4_^−^	Al(OH)_4_^−^	Al(OH)_4_^−^	Al(OH)_4_^−^	Zn^2+^	Zn^2+^
R.A.	99.4%	99.6%	99.45%	99.65%	34.3%	30.7%
Other dissolved species and R.A.	Al(OH)_3_^0^ (0.5%) Al(OH)_2_^+^ (0.04%)	Al(OH)_3_^0^ (0.33%) Al(OH)_2_^+^ (0.015%)	Al(OH)_3_^0^ (0.5%) Al(OH)_2_^+^ (0.035%)	Al(OH)_3_^0^ (0.33%) Al(OH)_2_^+^ (0.015%)	ZnCl^+^ (16.9%) ZnOHCl^0^ (14.0%) Zn(SO_4_)_2_^2−^ (8.1%)	ZnOHCl^0^ (19.9%) ZnCl^+^ (15.1%) Zn(SO_4_)_2_^2−^ (7.3%)

**Table 2:** Al, In, and Zn concentrations (µg/sampler) measured in the dissolved fraction using passive samplers. Each sampler is immersed for four days out of the 16 days of experimentation in tank control or equipped with Al-Zn-In-anode or Zn-anode. Mean ± standard deviation. d.l = detection limit.

µg/Sampler	Control (*n* = 4)	Al-Zn-In-Anode (*n* = 4)	Zn-Anode (*n* = 4)
Al	<d.l	148 ± 22	<d.l
In	<d.l	<d.l	<d.l
Zn	<d.l	<d.l	360 ± 125

**Table 3:** Concentrations (±SD) of aluminium, indium, or zinc (µg/g dry weight) measured in the digestive glands of *M. gigas* placed in control condition or exposed for 16 days to Al-Zn-In-anode or Zn-anode. Mean ± standard deviation. d.l = detection limit.

µg/g Dry Weight (Digestive Gland)	Control (*n* = 32)	Al-Zn-In-Anode (*n* = 32)	Zn-Anode (*n* = 32)
Al	39.9 ± 12.1	29 ± 12.9	28 ± 11.5
In	<d.l	<d.l	<d.l
Zn	1590 ± 459	1348 ± 164	1718 ± 271

**Table 4:** Significant metabolites modulation detected in the gills of *M. gigas* after chronic exposure for 16 days of the product of Zn-anodes dissolution. Metabolites are classified by family. The power of modulation is given by the multiplication factor (Zinc effect). The up arrows represent up-modulations, and the down-arrows are for down-modulations. The score represents the Shymanski classification.

Group	Metabolite	Mode	Retention Time (min)	Formula	Adduct	Monoisotopic Mass (Da)	Observed Mass *(m/z*)	Theoretical Mass *(m/z)*	Mass Error (ppm)	Score	Zn^2+^ Effect	
**Amino acids and derivatives**	Proline	Pos/ Neg	1.36/1.35	C_5_H_9_NO_2_	[M^+^H]^+^/[M^−^H]^−^	115.0633	116.0712/114.0553	116.0706/114.0561	5.4/7.0	1	0.8/0.7	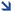
	Betaine	Pos	1.26	C_5_H_11_NO_2_	[M^+^K]^+^	117.0795	156.0424	156.0432	5.6	1	0.7	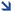
	Phenylalanine	Neg	6.52	C_9_H_11_NO_2_	[M^−^H]^−^	165.0795	164.0709	164.0722	8.1	1	0.8	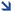
	Xanthurenic acid/Xanthurinate	Pos/ Neg	7.42/7.34	C_10_H_7_NO_4_	[M^+^H]^+^/Isotope of [M^−^CO_2_^−^H]^−^ at 160.0396	205.0375	206.0454/160.0396	206.0448/160.0404	3.2/5.2	2a	1.3/2.0	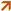
	Oxybetaine	Pos	1.29	C_6_H_14_NO^3+^	[M]^+^	148.0974	148.0971	148.0974	2.1	2a	0.7	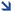
	Proline betaine (stachydrine)	Pos	1.21	C_7_H_14_NO^2+^	[M]^+^	144.1019	144.1025	144.1019	3.9	2a	0.7	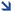
	Turicine	Pos	8.67	C_7_H_13_NO_3_	[M^+^H]^+^	159.0895	160.0968	160.0968	0.0	2a	0.8	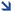
**Nucleotides and nucleosides**	Adenine	Pos/ Neg	6.79/6.74	C_5_H_5_N_5_	[M^+^H]^+^/[M^−^H]^−^	135.0545	136.0621/134.0463	136.0618/134.0472	2.5/6.5	2a	0.7/0.6	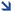
	Adenosine	Pos/ Neg	6.79/6.74	C_10_H_13_N_5_O_4_	[M^+^H]^+^/[M^−^H]^−^	267.0968	268.1046/266.0886	268.1040/266.0895	2.3/3.2	2a	0.8/0.6	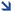
	Guanosine	Neg	6.52	C_10_H_13_N_5_O_5_	[M^−^H]^−^	283.0917	282.0839	282.0844	1.7	2a	1.2	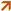
**Carnitine and derivatives**	Carnitine	Pos	1.18	C_7_H_16_NO^3+^	[M]^+^	162.1125	162.1130	162.1125	3.3	2a	0.8	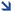
	3-dehydrocarnitine	Pos	1.33	C_7_H_16_NO^2+^	[M]^+^	146.1176	146.1179	146.1176	2.3	2a	0.8	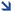
**Others**	Glycerophosphocholine	Pos	1.21	C_8_H_20_NO_6_P	[M^+^H]^+^	257.1028	258.1105	258.1101	1.7	2a	1.4	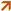
	Trigonelline (gynesine)	Pos	1.54	C_7_H_7_NO_2_	Isotope P^+2^ of [M^+^H]^+^	137.0477	138.0558	138.0550	6.1	2a	1.4	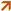
	Dodecanedioic acid	Neg	11.23	C_12_H_22_O_4_	[M-2H^+^Na]^−^	230.1518	251.1273	251.1265	3.2	2a	0.4	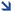
	Eicosanoid	Neg	10.85	C_20_H_32_O_5_	[M^−^H]^−^	352.2250	351.2172	351.2177	1.5	3	1.3	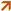

**Table 5:** Metabolites modulation detected in gills of *M. gigas* after chronic exposure for 16 days of the product of Al-Zn-In-anode dissolution. Metabolites are classified by family. The power of modulation is given by the multiplication factor (Al-Zn-In effect). The up arrows represent up-modulations and the down arrow are for down-modulations. The score represents the Shymanski classification.

Group	Metabolite	Mode	Retention Time (min)	Formula	Adduct	Monoisotopic Mass (Da)	Observed Mass *(m/z*)	Theoretical Mass *(m/z)*	Mass Error (ppm)	Score	Al-Zn-In Effect	
**Amino acids and derivatives**	Phenylalanine	Neg	6.52	C_9_H_11_NO_2_	[M^−^H]^−^	165.0795	164.0709	164.0722	0.8	1	0.8	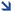
**Others**	Eicosanoid	Neg	10.85	C_20_H_32_O_5_	Isotope at 351.2166/[M^−^H]^−^	352.2250	351.2166	351.2177	3.1	3	1.3	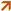

## Data Availability

Data is contained within the article.

## References

[1] Davy H. (1824). On the corrosion of copper sheeting by sea water, and on methods of preventing this effect; and on their application to ships of war and other ships. Philos. Trans. R. Soc. Lond..

[2] Lemieux E., Hartt W.H., Lucas K.E. A critical review of Al anode activation and dissolution mechanisms and performance. Proceedings of the Paper NACE-01509, CORROSION 2001 Conference.

[3] Muñoz A.G., Saidman S.B., Bessone J.B. (2002). Corrosion of an Al–Zn–In alloy in chloride media. Corros. Sci..

[4] Ma J., Wen J., Zhai W., Li Q. (2012). In situ corrosion of Al-Zn-In-Mg-Ti-Ce sacrificial anode alloy. Mater. Charac..

[5] Caplat C., Oral R., Mahaut M.-L., Mao A., Barillier D., Guida M., Della Rocca C., Pagano G. (2010). Comparative toxicities of aluminum and zinc from sacrificial anodes or from sulfate salt in sea urchin embryos and sperm. Ecotoxicol. Environ. Saf..

[6] Muttin E., Caplat C., Latire T., Mottier A., Mahaut M.-P., Costil K., Barillier D., Lebel J.-M., Serpentini A. (2012). Effect of zinc sacrificial anode degradation on the defence system of the Pacific oyster, *Crassostrea gigas*: Chronic and acute exposures. Mar. Pollut. Bull..

[7] Barbarin M., Turquois C., Dubillot E., Huet V., Churlaud C., Muttin F., Thomas H. (2023). First quantitative biomonitoring study of two ports (marina, commerce) in French littoral area: Evaluation of metals released into the marine environment and resulting from galvanic anodes. Sci. Total Environ..

[8] Levallois A., Caplat C., Basuyaux O., Lebel J.-M., Laisney A., Costil K., Serpentini A. (2022). Effects of chronic exposure of metals released from the dissolution of an aluminium galvanic anode on the Pacific oyster *Crassostrea gigas*. Aquat. Toxicol..

[9] Levallois A., Nivelais L., Caplat C., Lebel J.-M., Basuyaux O., Costil K., Serpentini A. (2023). Impact assessment of metals realeased by aluminium-based galvanic anode on the physiology of the abalone *Haliotis tuberculata* in controlled conditions. Ecotoxicology.

[10] Breitwieser M., Vigneau E., Viricel A., Becquet V., Lacroix C., Erb M., Huet V., Churlaud C., Le Floch S., Guillot B. (2018). What is the relationship between the bioaccumulation of chemical contaminants in the variegated scallop *Mimachlamys varia* and its health status? A study carried out on the French Atlantic coast using the Path ComDim model. Sci. Total Environ..

[11] Gibson G. Behavior of Al-Zn-In anodes at elevated temperature. Proceedings of the Paper NACE-10369, CORROSION 2010 Conference.

[12] Parkhurst D.L., Appelo C.A.J. (1999). User’s guide to PHREEQC (Version 2)—A computer program for speciation, batch-reaction, one-dimensional transport, and inverse geochemical calculations: U.S. Geological Survey. Water-Resour. Investig. Rep..

[13] Allison J.D., Brown D.S., Novo-Gradac K.J. (1990). MINTEQA2/PRODEFA2—A Geochemical Assessment Model for Environmental Systems—Version 3.0 User’s Manual.

[14] US Environmental Protection Agency (1998). MINTEQA2/PRODEFA2, A Geochemical Assessment Model for Environmental Systems—User Manual Supplement for Version 4.0.

[15] (2021). Standard Practice for Preparation of Substitute Ocean Water.

[16] Breitwieser M., Viricel A., Graber M., Murillo L., Becquet V., Churlaud C., Fruitier-Arnaudin I., Huet V., Lacroix C., Pante E. (2016). Short-Term and Long-Term Biological Effects of Chronic Chemical Contamination on Natural Populations of a Marine Bivalve. PLoS ONE.

[17] Miramand P., Bustamante P., Bentley D., Kouéta N. (2006). Variation of heavy metal concentrations (Ag, Cd, Co, Cu, Fe, Pb, V, and Zn) during the life cycle of the common cuttlefish *Sepia officinalis*. Sci. Total Environ..

[18] Metian M., Warnau M., Oberhansli F., Teyssie J.L., Bustamante P. (2007). Interspecific comparison of Cd bioaccumulation in European Pectinidae (*Chlamys varia* and *Pecten maximus*). J. Exp. Mar. Biol. Ecol..

[19] Deborde J., Refait P., Bustamante P., Caplat C., Basuyaux O., Grolleau A.M., Mahaut M.L., Brach-Papa C., Gonzalez J.L., Pineau S. (2015). Impact of Galvanic Anode Dissolution on Metal Trace Element Concentrations in Marine Waters. Water Air Soil Pollut..

[20] Ory P., Hamani V., Bodet P.E., Murillo L., Graber M. (2021). The variegated scallop, *Mimachlamys varia*, undergoes alterations in several of its metabolic pathways under short-term zinc exposure. Comp. Biochem. Physiol. Part D Genom. Proteom..

[21] Mondeguer F., Abadie E., Herve F., Bardouil M., Sechet V., Raimbault V., Berteaux T., Zendong S.Z., Palvadeau H., Amzil Z. (2015). Pinnatoxines en Lien Avec L’espèce Vulcanodinium rugosum (II). http://archimer.ifremer.fr/doc/00285/39635/.

[22] Ory P., Bonnet A., Mondeguer F., Breitwieser M., Dubillot E., Graber M. (2019). Metabolomics based on UHPLC-QToF- and APGCQToF-MS reveals metabolic pathways reprogramming in response to tidal cycles in the sub-littoral species *Mimachlamys varia* exposed to aerial emergence. Comp. Biochem. Physiol. Part D Genom. Proteom..

[23] Van Der Kloet F.M., Bobeldijk I., Verheij E.R., Jellema R.H. (2009). Analytical error reduction using single point calibration for accurate and precise metabolomic phenotyping. J. Proteome Res..

[24] Dührkop K., Fleischauer M., Ludwig M., Aksenov A.A., Melnik A.V., Meusel M., Dorrestein P.C., Rousu J., Böcker S. (2019). SIRIUS 4: A rapid tool for turning tandem mass spectra into metabolite structure information. Nat. Methods.

[25] Schymanski E.L., Jeon J., Gulde R., Fenner K., Ruff M., Singer H.P., Hollender J. (2014). Identifying small molecules via high resolution mass spectrometry: Communicating confidence. Environ. Sci. Technol..

[26] Gensemer R.W., Playle R.C. (1999). The bioavailability and toxicity of aluminum in aquatic environments. Crit. Rev. Environ. Sci. Technol..

[27] Breitwieser M., Barbarin M., Huet V., Dubillot E., Graber M., Thomas H., Muttin F., Thomas H. (2021). Comparative biomarkers study in two scallop organs to establish guidelines for evaluating French Atlantic coastline water quality. Marine Environmental Quality: Healthy Coastal Waters.

[28] Trevisan R., Mello D.F., Delapedra G., Silva D.G.H., Arl M., Danielli N.M., Metian M., Almeida E.A., Dafre A.L. (2016). Gills as a glutathione-dependent metabolic barrier in Pacific oysters *Crassostrea gigas*: Absorption, metabolism and excretion of a model electrophile. Aquat. Toxicol..

[29] Caplat C., Mottin E., Lebel J.-M., Serpentini A., Barillier D., Mahaut M.-L. (2012). Impact of a Sacrificial Anode as Assessed by Zinc Accumulation in Different Organs of the Oyster *Crassostrea gigas*: Results from Long- and Short-Term Laboratory Tests. Arch. Environ. Contam. Toxicol..

[30] Mao A., Mahaut M.-L., Pineau S., Barillier D. (2011). Assessment of sacrificial anode impact by aluminum accumulation in mussel *Mytilus edulis*: A large-scale laboratory test. Mar. Pollut. Bull..

[31] Kirchgeorg T., Weinberg I., Hörnig M., Baier R., Schmid M.J., Brockmeyer B. (2018). Emissions from corrosion protection systems of offshore wind farms: Evaluation of the potential impact on the marine environment. Mar. Pollut. Bull..

[32] Yang J.-L. (2014). Comparative acute toxicity of gallium(III), antimony(III), indium(III), cadmium(II), and copper (II) on freshwater swamp shrimp (*Macrobrachium nipponense*). Biol. Res..

[33] Séguin A., Caplat C., Serpentini A., Lebel J.-M., Menet-Nedelec F., Costil K. (2016). Metal bioaccumulation and physiological condition of the Pacific oyster (*Crassostrea gigas*) reared in two shellfish basins and a marina in Normandy (northwest France). Mar. Pollut. Bull..

[34] Ivanina A.V., Cherkasov A.S., Sokolova I.M. (2008). Effects of cadmium on cellular protein and glutathione synthesis and expression of stress proteins in eastern oysters, *Crassostrea virginica* Gmelin. J. Exp. Biol..

[35] Sokolova I.M., Lannig G. (2008). Interactive effects of metal pollution and temperature on metabolism in aquatic ectotherms: Implications of global climate change. Clim. Res..

[36] Sokolova I.M., Frederich M., Bagwe R., Lannig G., Sukhotin A.A. (2012). Energy homeostasis as an integrative tool for assessing limits of environmental stress tolerance in aquatic invertebrates. Mar. Environ. Res..

[37] Meng J., Wang W.-X., Li L., Zhang G. (2017). Respiration disruption and detoxification at the protein expression levels in the Pacific oyster (*Crassostrea gigas*) under zinc exposure. Aquat. Toxicol..

[38] Liu X., Sun H., Wang Y., Ma M., Zhang Y. (2014). Gender-specific metabolic responses in hepatopancreas of mussel *Mytilus galloprovincialis* challenged by *Vibrio harveyi*. Fish Shellfish. Immunol..

[39] Ramirez G., Gomez E., Dumas T., Rosain D., Mathieu O., Fenet H., Courant F. (2022). Early Biological Modulations Resulting from 1-Week Venlafaxine Exposure of Marine Mussels *Mytilus galloprovincialis* Determined by a Metabolomic Approach. Metabolites.

[40] Lucena M.N., Garçone D.P., Fontes C.F.L., Fabria L.M., Moraes C.M., McNamara J.C., Leone F.A. (2019). Dopamine binding directly up-regulates (Na+, K+)-ATPase activity in the gills of the freshwater shrimp *Macrobrachium amazonicum*. Comp. Biochem. Physiol. Part A.

[41] Carroll M.A., Catapane E.J. (2007). The nervous system controls of lateral ciliary activity of the gill of the bivalve mollusc, *Crassostrea virginica*. Comp. Biochem. Physiol. A Mol. Integr. Physiol..

[42] Aru V., Sarais G., Savorani F., Engelsen S.B., Cesare Marincola F. (2016). Metabolic responses of clams, *Ruditapes decussatus* and *Ruditapes philippinarum*, to short-term exposure to lead and zinc. Mar. Pollut. Bull..

[43] Christgen S.L., Becker D.F. (2019). Role of Proline in Pathogen and Host Interactions. Antioxid. Redox Signal..

[44] Tikunov A.P., Johnson C.B., Lee H., Stoskopf M.K., Macdonald J.M. (2010). Metabolomic Investigations of American Oysters Using 1H-NMR Spectroscopy. Mar. Drugs.

[45] Zhou C., Song H., Feng J., Hu Z., Yang M.J., Shi P., Guo Y.J., Li H.Z., Zhang T. (2022). Metabolomics and biochemical assays reveal the metabolic responses to hypo-salinity stress and osmoregulatory role of cAMP-PKA pathway in *Mercenaria mercenaria*. Comput. Struct. Biotechnol. J..

[46] Nikitashina V., Stettin D., Pohnert G. (2022). Metabolic adaptation of diatoms to hypersalinity. Phytochemistry.

[47] Cao C., Wang W.-X. (2016). Bioaccumulation and metabolomics responses in oysters *Crassostrea hongkongensis* impacted by different levels of metal pollution. Environ. Pollut..

[48] Ma L., Lu J., Yao T., Ye L., Wang J. (2021). Gender-Specific Metabolic Responses of *Crassostrea hongkongensis* to Infection with *Vibrio harveyi* and Lipopolysaccharide. Antioxidants.

[49] Frizzo R., Bortoletto E., Riello T., Leanza L., Schievano E., Venier P., Mammi S. (2021). NMR Metabolite Profiles of the Bivalve Mollusc *Mytilus galloprovincialis* Before and After Immune Stimulation With *Vibrio splendidus*. Front. Mol. Biosci..

[50] Ji C., Wang Q., Wua H., Tan Q., Wang W.-X. (2015). A metabolomic investigation of the effects of metal pollution in oysters *Crassostrea hongkongensis*. Mar. Pollut. Bull..

[51] Gallazzini M., Burg M.B. (2009). What’s New About Osmotic Regulation of Glycerophosphocholine. Physiology.

[52] Burg M.B., Ferraris J.D. (2008). Intracellular Organic Osmolytes: Function and Regulation. J. Biol. Chem..

[53] Fuhrman M., Delisle L., Petton B., Corporeau C., Pernet F. (2018). Metabolism of the Pacific oyster, *Crassostrea gigas*, is influenced by salinity and modulates survival to the Ostreid herpes virus OsHV-1. Biol. Open.

[54] Ivanina A.V., Sokolova I.M. (2013). Interactive effects of pH and metals on mitochondrial functions of intertidal bivalves *Crassostrea virginica* and *Mercenaria mercenaria*. Aquat. Toxicol..

[55] Handy D.E., Loscalzo J. (2012). Redox Regulation of Mitochondrial Function. Antioxid. Redox Signal..

[56] Gülçin I. (2006). Antioxidant and antiradical activities of l-carnitine. Life Sci..

[57] Späth J., Fick J., McCallum E., Cerveny D., Nording M.L., Brodin T. (2022). Wastewater effluent affects behaviour and metabolomic endpoints in damselfly larvae. Sci. Rep..

[58] Longo N., Frigeni M., Pasquali M. (2016). Carnitine transport and fatty acid oxidation. Biochim. Biophys. Acta.

[59] Fu Q., Scheidegger A., Laczko E., Hollender J. (2021). Metabolomic Profiling and Toxicokinetics Modeling to Assess the Effects of the Pharmaceutical Diclofenac in the Aquatic Invertebrate *Hyalella azteca*. Environ. Sci. Technol..

[60] Hamani V., Ory P., Bodet P.-E., Murillo L., Graber M. (2021). Untargeted Metabolomics Reveals a Complex Impact on Different Metabolic Pathways in Scallop *Mimachlamys varia* (Linnaeus, 1758) after Short-Term Exposure to Copper at Environmental Dose. Metabolites.

[61] Léonard J.A., Cope W.G., Barnhart M.C., Bringolf R.B. (2014). Metabolomic, behavioral, and reproductive effects of the aromatase inhibitor fadrozole hydrochloride on the unionid mussel *Lampsilis fasciola*. Gen. Comp. Endocrinol..

[62] Ferdinandusse S., Denis S., van Roermund C.W.T., Wanders R.J.A., Dacremont G. (2004). Identification of the peroxisomal β-oxidation enzymes involved in the degradation of long-chain dicarboxylic acids. J. Lipid Res..

[63] Watanabe M., Meyer K.A., Jackson T.M., Schock T.B., Johnson W.E., Bearden D.W. (2015). Application of NMR-based metabolomics for environmental assessment in the Great Lakes using zebra mussel (*Dreissena polymorpha*). Metabolomics.

[64] Cappello T., Maisano M., Mauceri A., Fasulo S. (2017). 1 H NMR-based metabolomics investigation on the effects of petrochemical contamination in posterior adductor muscles of caged mussel *Mytilus galloprovincialis*. Ecotoxicol. Environ. Saf..

[65] Dumas T., Bonnefille B., Gomez E., Boccard J., Ariza Castro N., Fenet H., Courant F. (2020). Metabolomics approach reveals disruption of metabolic pathways in the marine bivalve *Mytilus galloprovincialis* exposed to a WWTP effluent extract. Sci. Total Environ..

[66] De Sotto R.B., Medriano C.D., Cho Y., Kim H., Chung I.-Y., Seok K.-S., Hong S.W., Park Y., Kim S. (2017). Sub-lethal pharmaceutical hazard tracking in adult zebrafish using untargeted LC–MS environmental metabolomics. J. Hazard. Mater..

[67] Canesi L., Miglioli A., Balbi T., Fabbri E. (2022). Physiological Roles of Serotonin in Bivalves: Possible Interference by Environmental Chemicals Resulting in Neuroendocrine Disruption. Front. Endocrinol..

[68] Monmai C., Go S.H., Shin I.S., You S.G., Lee H., Kang S.B., Park W.J. (2018). Immune-Enhancement and Anti-Inflammatory Activities of Fatty Acids Extracted from *Halocynthia aurantium* Tunic in RAW264.7 Cells. Mar. Drugs.

